# Working memory capacity and self‐cues: Consistent benefits in children and adults

**DOI:** 10.1111/bjop.12778

**Published:** 2025-02-14

**Authors:** Zahra Ahmed, Janet F. McLean, Kevin Allan, Sheila J. Cunningham

**Affiliations:** ^1^ Faculty of Social and Applied Sciences, Kydd Building Abertay University Dundee UK; ^2^ School of Health in Social Science, Old Medical School University of Edinburgh Edinburgh UK; ^3^ School of Psychology, William Guild Building University of Aberdeen Aberdeen UK

**Keywords:** binding, capacity, self‐cues, self‐prioritization, working memory

## Abstract

From attentional prioritization to enhanced memory, self‐cues trigger a variety of effects within human cognition. Recent work suggests that self‐reference may also enhance working memory, possibly via attentional prioritization. However, there is no direct evidence that self‐cues enhance working memory capacity, or that such boosts covary with individuals' attentional function. Here, we provide the first direct evidence of enhanced working memory capacity for self‐referential cues, independent of attentional processing. We adapted a verbal working memory complex span to create a ‘Self’ condition (featuring the participant's own name), ‘Other’ condition (featuring a non‐self‐name), and Control condition (with no name), in 7–9‐year‐old children (Exp.1, *N* = 71) and adults (Exp.2, *N* = 52). In both experiments, the Self condition elicited significantly higher spans than the other conditions (Exp 1: *p* < .001, *η*_*p*_^2^ = .32; Exp 2: *p* < .001, *η*_*p*_^2^ = .25), but this increase in capacity was unrelated to measures of attentional processing or backward digit span. Moreover, equivalent boosts were observed in children and adults, despite adults' significantly higher underlying capacity. We propose a chunking interpretation based on enhanced binding of self‐associated items, directly benefiting individual's working memory capacity regardless of their current attentional competence or ‘baseline’ capacity.

## INTRODUTION

The limited capacity of working memory both within and across domains means that its processing is heavily influenced by selective attention and the consequent prioritization of some stimuli over others (see Myers et al., 2017). Stimuli associated with attentional priority such as self‐cues (e.g. one's own name) might be expected to be prioritized in working memory, facilitating the processing of self‐referent content. However, the impact of self‐cues on core indices of working memory function has never been directly demonstrated, despite assumptions about its impact playing an active role in the interpretation of various empirical findings (e.g. D'Ailly et al., 1997; Golubickis & Macrae, 2023; Yin et al., 2019). Mnemonic strategies, such as chunking, are well‐established methods for enhancing working memory capacity by organizing discrete items into integrated units for more efficient encoding and retrieval (Cowan, 2001; Miller, 1956; Thalmann et al., 2019). Whilst self‐cues are hypothesized to enhance working memory through mechanisms such as attentional prioritization, it remains unclear whether this process overlaps with strategies like chunking. Here, for the first time, we test whether self‐cues do in fact ease a fundamental constraint on working memory function: its capacity. We investigate the effects of self‐cues on working memory capacity in middle childhood where this variable is linked with educational attainment, and in adulthood when the underlying capacity of working memory is larger.

The current study utilizes verbal working memory tasks to investigate capacity. The partition of working memory capacity into verbal and visuo‐spatial domains reflects roles outlined in Baddeley and Hitch's seminal model (Baddeley, 2000; Baddeley & Hitch, 1974). This proposes domain‐specific storage systems independently responsible for verbal‐acoustic and visuo‐spatial material respectively, and a domain‐general executive component responsible for allocating attentional resources and interacting with long‐term memory (Baddeley et al., 2020). Common approaches to exploring the nature of working memory capacity include comparing memory performance during single and dual tasks and examining performance on complex span tasks such as the reading span task (Daneman & Carpenter, 1980) and the operation span task (Turner & Engle, 1989). Whilst complex span tasks may require a domain‐specific modality for coding and rehearsal of the material, the evidence suggests that all complex span tasks reflect a domain‐general capacity (see Kane et al., 2004).

The limited capacity of working memory both within and across domains means that processing is driven by attentional priority, as attention needs to be selectively focused on important information whilst distracting information is ignored (see Myers et al., 2017). Self‐referent cues have been shown to be prioritized across verbal and visual domains, including familiar cues like participants' own names (e.g. Moray, 1959; Röer et al., 2013) as well as newly learned self‐associations (e.g. shape‐referent pairings, Sui et al., 2012; external voices, Payne et al., 2021). Based on these studies, it has been argued that self‐cues are influential across all three of Peterson and Posner's (2012) proposed facets of attention: alerting, orienting and executive control (Sui & Rotshtein, 2019). First, alertness can be increased by self‐cues via a norepinephrine‐based physiological response, perhaps an early conditioned response to the sound of one's own name precipitating an expected behaviour (Kaida & Abe, 2018). In dull tasks like extended vigilance, performance significantly improves when a participant's own name is repeatedly presented (relative to presentation of a non‐word comprising the same syllable sounds, a beep, or no sound), indicating an effect of self‐cues on alertness (Kaida & Abe, 2018; see also Kaida & Iwaki, 2018). Second, own‐name cues elicit attention orienting responses; the classic ‘cocktail party effect’ (Cherry, 1953), whereby attention can be focused intentionally on one of two audio channels in a dichotic listening task, is disrupted by presentation of a participant's own name in the unattended channel (Moray, 1959). A large body of research speaks to the robustness of the attentional orienting effects of verbal self‐cues (see Bargh, 1982; Röer et al., 2013; Wood & Cowan, 1995) from a very young age (Imafuku et al., 2014). Finally, there is evidence that self‐cues are influential at an executive level of attention, for example, eliciting enhanced intentional inhibition of self‐relative to other‐associated stimuli (although research here is limited to visual cues—see Golubickis et al., 2021; Svensson et al., 2023). It seems verbal and visual self‐cues serve the adaptive purpose of alerting the cognitive system that something in the environment is personally relevant, and too important to be missed.

A key question is whether cues that are prioritized in attention impact on working memory capacity directly. One potential mechanism is that automatic prioritization may reduce working memory load by mitigating the need for executive control of attentional focus (Oberauer, 2019). Once active in working memory storage, prioritized items like self‐cues are associated with increased binding (e.g. Huang et al., 2023; Sui & Humphreys, 2015; Wang et al., 2016), which facilitates working memory storage as items are held as integrated representations in the episodic buffer (Hitch et al., 2020). If the quantity of chunks held in verbal WM is fixed (i.e. at about four items; Cowan, 2001; Miller, 1956) but the quantity of information within chunks can vary, then binding promoted by self‐cues should increase storage potential. Supporting this reasoning, in a visuo‐spatial task, Yin et al. (2019) trained participants to associate a set of referent labels (e.g. self, stranger) with specific colours, then presented pairs of circles in these colours. Across four experiments, they found that participants' location matching responses were reliably faster for circles in self‐associated colours than those associated with other referents, suggesting they were prioritized in working memory. Accuracy was at ceiling, so storage was not assessed directly; however, it seems reasonable to interpret faster responses to self‐associated cues in terms of working memory either as an indicator of either more effective binding of the cue with the label, or more capacity for processing the shape‐label pairings in working memory. Yin et al. deliberately employed arbitrary self‐associations to reduce the influence of familiar cues (see Golubickis & Macrae, 2023). However, the use of familiar cues like own‐name cues is likely to be particularly powerful as familiar information is more easily stored in WM, perhaps as a result of long‐term memory providing some protection from trace decay (Jackson & Raymond, 2008), or as a result of the WM system using activated long‐term memory constructs as ‘outsourced’ storage (Bartsch & Shepherdson, 2022; Ngiam, 2024; Ngiam et al., 2019). Given the sparsity of evidence relating self‐cue presentation to verbal working memory, the current study investigated the effects of familiar own‐name cues on capacity.

Interestingly, there is indirect evidence from mathematical processing tasks that verbal self‐cues can increase verbal working memory capacity in children. D'Ailly et al. (1997) employed the personal pronoun ‘you’ in verbal maths word problems, showing that this manipulation facilitated students' speed and accuracy relative to questions in which ‘you’ was replaced by another character's name (see also Cunningham et al., 2024). Whilst not directly testing the effects of self‐cues on working memory capacity, these findings show enhanced performance on a verbal task in which errors often reflect working memory capacity limits (Lemaire, 1996), consistent with the predicted effects of self‐cues on capacity.

D'Ailly et al.'s study was conducted with children, who tend to have lower working memory capacity than adults (Reynolds et al., 2022). This is important because any facilitative effects of self‐cues on working memory span may be particularly strong in conditions of reduced capacity. Indeed, it has been reported that attention switching in response to one's own name is stronger in participants with low working memory capacity, who are less able to inhibit attention capture by external cues (Conway et al., 2001; Conway & Kane, 2001). However, research on the effects of self‐cues on cognition more broadly has reported surprisingly little individual variation across individuals, suggesting attentional effects of self‐cues may be independent of underlying cognitive differences, including age‐related development. In particular, attentional self‐prioritization effects remain stable across middle childhood (Maire et al., 2020) and between childhood and adulthood (Singh & Karnick, 2022), and age‐invariant ‘incidental’ self‐reference effects have been widely reported in long‐term memory (e.g. Andrews et al., 2020; Cunningham et al., 2014; Dunbar et al., 2016; Hutchison et al., 2021). If these effects are driven by enhanced binding of self‐associated items, participants of all working memory capacity should benefit with relative consistently (e.g. see Huntley et al., 2011).

The issue of whether any effects of self‐cues on working memory would vary across individuals and across development is worth investigating for both theoretical and practical reasons. From a theoretical perspective, the impact of self‐cues on working memory capacity is a notable gap in our understanding of the cognitive consequences of prioritized stimuli. Practically, it is of real‐world interest to reduce working memory load for children in educational contexts (e.g. see Sweller, 1988) and for adults dealing with occupational and everyday life tasks with a high working memory load. If self‐cues are more effective at supporting working memory in those with lower capacity (as working memory research would suggest; Conway et al., 2001; Conway & Kane, 2001), then self‐referential strategies should be targeted at individuals with lower working memory capacity, for example, towards children rather than adults. In contrast, if the effects are not bound by individual differences in working memory but follow the pattern of working memory strategies like chunking, such strategies should be consistently effective. It is therefore important to examine the effects of self‐cues across individual differences in attention and working memory, and across childhood to adulthood.

The current study tested whether the inclusion of a participant's own name increases complex verbal working memory (VeWM) span in both children (7–9 year‐olds, Experiment 1) and adults (Experiment 2). VeWM span was measured using an adapted listening span task (Daneman & Carpenter, 1980), in which participants hear a set of short sentences and are asked to indicate whether each one either makes sense or is a nonsense sentence. After each set (e.g. of three sentences) is complete, participants are asked to recall the last word of each sentence in the set. The longest set from which participants can correctly recall the last words is taken as their VeWM span. This task is designed to assess both storage and processing, as performance is related to the updating and monitoring of incoming information as well as short‐term maintenance and is therefore useful test of overall capacity (see Miyake et al., 2000).

We adapted the listening span task to construct three referent conditions. In the ‘Self’ condition, each sentence included the participant's own name, in the ‘Other’ condition, the sentence included a stranger's name, and in the ‘Control’ condition, the sentence did not include any names. An additional feature of the study was the inclusion of standard cognitive measures of working memory and attention, to examine whether these were related to any effect of self on VeWM span. Specifically, we measured forward and backward digit span along with performance on measures of selective attention, attentional control, and sustained attention. Experiment 1 was conducted with children and Experiment 2 with adults; both experiments were pre‐registered using the As predicted template, which can be accessed at https://aspredicted.org/BTX_9G8 (Experiment 1), and https://aspredicted.org/1XW_L9V (Experiment 2).

## EXPERIMENT 1: OWN‐NAME EFFECTS ON CHILDREN'S VeWM CAPACITY

The existing research linking self‐cues to VeWM task performance was conducted with children as young as 7 years old and involved verbal presentation of numerical problem‐solving questions (D'Ailly et al., 1997). As this choice of task suggests, one reason for exploring the effects of self‐cues on processing in childhood is that VeWM processing is inherent across multiple educational activities, so is particularly important to understand at this stage. Further, there is a growing body of evidence that the cognitive effects of self‐bias are highly robust across childhood in both perception (Maire et al., 2020; Singh & Karnick, 2022) and long‐term memory (Andrews et al., 2020; Cunningham et al., 2013, 2014; Hutchison et al., 2021; Ross et al., 2011, 2020). Indeed, even 5‐month‐old infants have been shown to produce different ERP signals in response to own‐name and other‐name cue presentation (Parise et al., 2010). It would therefore be expected that if there are effects of self‐cues on working memory capacity, these should be detectable in childhood.

Experiment 1 tested the effects of own‐name inclusion on participant's VeWM span in children aged 7–9 years. It was hypothesized that VeWM would be higher in the ‘Self’ condition (in which the sentences presented to children included their own name) than the Other condition (including a stranger's name) or Control condition (with no names). No differences were expected between the Other and Control conditions. To examine the extent to which any increase in working memory capacity in the ‘Self’ condition varied across individual differences in working memory or attentional abilities, we also included standard measures of working memory storage (forwards digit span), storage and processing (backward digit span), and attentional processing (selective attention, attention control [also known as attentional switching], and sustained attention subtests of the Tests of Everyday Attention for Children [TEA‐Ch], Manly et al., 1999).

### Method

#### Participants

Seventy‐one participants (36 female) took part in the study. The ages ranged from 7 to 9 years (*M* = 8.47, SD = .84). Participants were recruited from a Scottish primary school with a predominantly un‐deprived catchment area. Children were tested over two sessions after written consent from parents and approval from the headteacher. Due to constraints arising from the 2020 COVID‐19 pandemic, 19 participants were unable to attend the second testing session, missing one attention measure as a result; otherwise, all children completed all tasks. The experiment was approved by Abertay University's Research Ethics Committee.

#### Materials and procedure

Participants were tested individually across two sessions in a quiet location at their school. In the first session, the children completed two Digit Span tasks followed by the VeWM test and two TEA‐Ch subtests: *Map Mission* and *Opposite Worlds*. The final TEA‐Ch subtest (*Score!*) was administered in the second session, when participants also completed a numerical problem‐solving task reported elsewhere (Cunningham et al., 2024—pilot study; includes digit span and TEA‐Ch data from a subset of current participants).

The two Digit Span tasks comprised the Digit Forwards test and Digit Backwards test from the Wechsler Intelligence Scale for Children‐Third Edition (WISC‐III: Wechsler, 1991). Both the Digit Forwards and Digit Backwards test began with two trials per set, proceeding to a maximum of nine digits in the Digit Forwards test and eight in the Digit Backwards test. In each trial, items are presented verbally, and the participant is required to repeat them verbatim (Digit Forward test) or in reverse order (Digit Backwards test). Participants could attempt each number of digits up to two times; the test ended if the child failed both trials. For each trial, participants received a score of either 0 or 1, with a maximum achievable score of 16 for the Digit Forwards test, and 14 for the Digit Backwards test.

The VeWM task was based on the listening recall span task developed by Daneman and Carpenter (1980) and was adapted to include Self, Other, and Control conditions. Participants were presented with sets of sentences, ranging from two to nine sentences per set. The experimenter explained that sentences would be read out aloud and asked the participant to do two things: determine whether each sentence made sense, and keep the last word in mind so that they could repeat all of the last words, in any order, at the end of the set. An example of a three‐sentence Other set is shown below, with the correct participant response in parentheses:

- ‘Sam drank his orange computer’ (‘No’)
- ‘Sam ran fast and won the race’ (‘Yes’)
- ‘Sam went to the cinema to watch a house’ (*‘*No’)

The child would ‘pass’ this three‐sentence set if they were able to recall the words ‘computer, race, house’ (in any order) at the end of the set.

Three lists of sentences were produced, each containing 132 test sentences plus three sets of two‐sentence practice sets. Within each list, there were 27 sets ranging from two to nine trial sentences. The number of words within sentences was consistent across the conditions, ranging from 5 to 12 words. Participants could attempt each set up to three times; the test ended if the child failed all three attempts. The highest set recalled without three failures was recorded as the participant's verbal WM span.

The sets included both nonsense and non‐nonsense sentences to ensure participants were processing the entire sentence rather than just focusing on the target word. The number of nonsense and non‐nonsense sentences within the sets was consistent across conditions. To adapt lists for the Self and Other conditions, the experimenter verbalized either the participant's name (Self condition) or the gender‐neutral name ‘Sam’ (Other condition) at the appropriate point, with pronouns indicating a female Sam for male participants and male Sam for female participants to minimize self‐projection (Cunningham et al., 2014). The Self and Other conditions were counterbalanced so that for half of participants List 1 was used for Self, and in the other half, List 1 included the Other‐referent. List 3 consisted of control sentences without any specific referent, such as ‘It was very windy, so the bridge was closed’. The order of conditions (Self, Other, Control) was counterbalanced across participants to mitigate any potential influence of practice effects.

Participants completed three subtests of attention from the TEA‐Ch. Selective attention was assessed with *Map Mission*. Participants were presented with a laminated map of Philadelphia, USA. The map comprised various similar‐sized symbols, and participants were instructed to locate and circle with a pen as many target symbols (a knife and fork dining symbol) as they could within 1 min. Participant's score was the number of correctly identified symbols, with a total possible count of 80. Participants then completed *Opposite Worlds*, a measure of attention control. This involved following four different paths containing numbers ‘1’ and ‘2’. In one version, *Same World*, the participants read aloud the numbers verbatim. In *Opposite World*, however, children were instructed to say ‘2’ when they saw ‘1’ and ‘1’ when they saw ‘2’. Completion time to finish each path correctly was recorded. Participants' score was the total time for each world, calculated by adding the two paths for *Same World* and two paths for *Opposite World*, with faster completion times indicating better performance. Lastly, to measure sustained attention, participants completed a task called *Score!*. Wearing headphones, the children listened to a series of laser sounds played from a CD over 10 trials. Participants were required to keep track of the number of laser sounds played, similar to keeping score in a video game, and report the number verbally to the experimenter after each trial. The sounds were presented at irregular intervals within each trial to assess participants' ability to sustain attention, and the children were instructed not to use their fingers to keep track. The number of correctly recalled trials out of 10 were recorded.

### Results

Two measures of working memory capacity were analysed: a Working Memory Span, and an Attempt score. Attempt scores were calculated for each participant as an additional measure of WM because spans typically have a narrow range and low variability, potentially reducing our ability to detect relationships with other variables (i.e. age and performance on working memory and attention tasks).

#### Verbal working memory span

**FIGURE 1:**
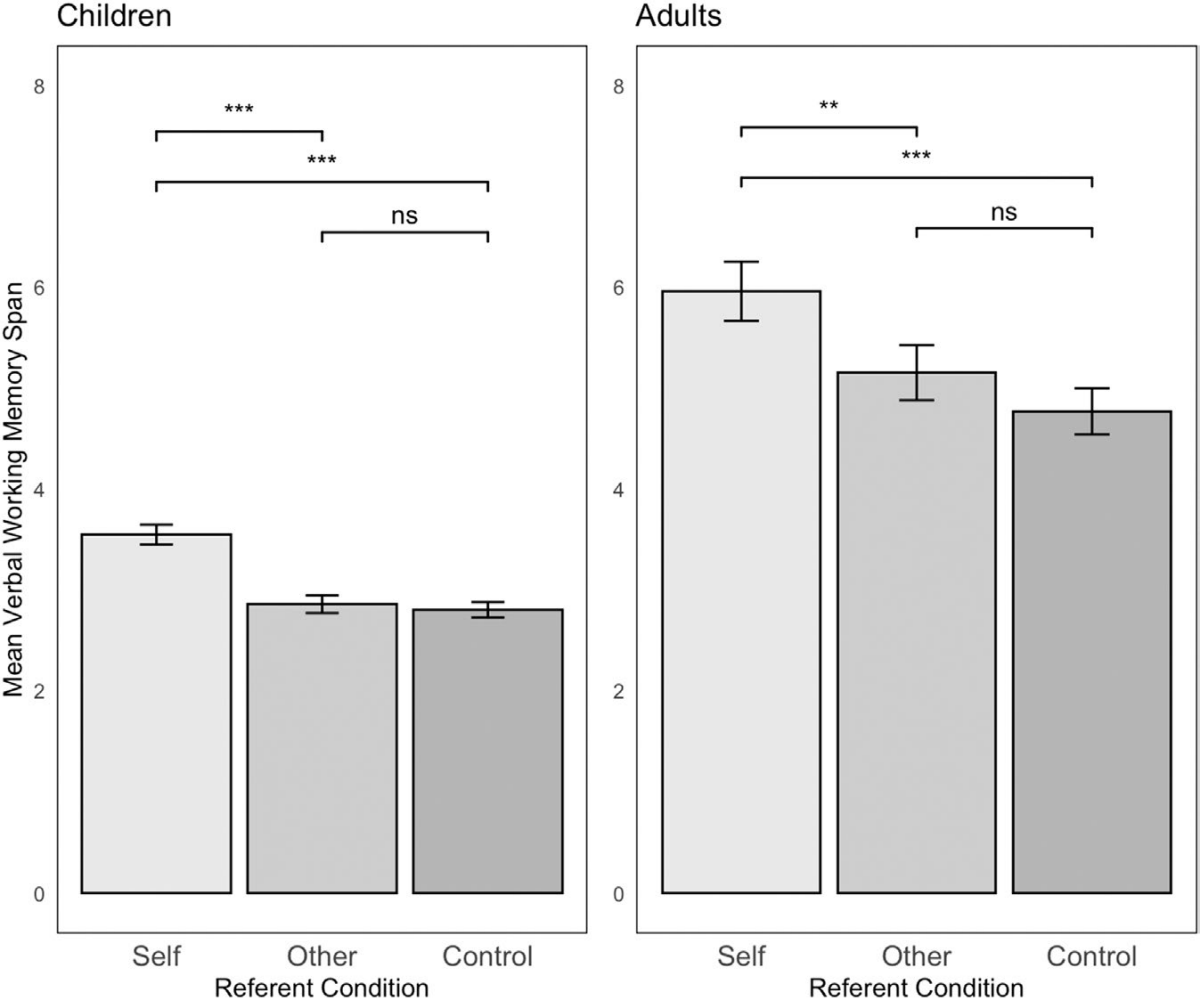
Mean verbal working memory span for Self, Other, and Control trials across Experiments 1 (children) and 2 (adults). ***p < .001, **p < .01.

The number of sentences included in the highest set correctly recalled by each participant was recorded as their VeWM span. Figure 1 shows the mean VeWM spans in the Self (Range = 4, Max = 6), Other (Range = 3, Max = 5), and Control (Range = 3, Max = 5) conditions. A repeated measures ANOVA^1^ revealed a significant effect of Referent (Self, Other, Control) on VeWM span, *F* (2, 140) = 33.37, *p* < .001, *η*_*p*_^2^ = .32. Pairwise comparisons between the three conditions of Referent were conducted with a Tukey adjustment. The comparisons showed that participants had higher spans in the Self condition (i.e. when the sentences contained their own name), *M* = 3.55, 95% CI [3.35, 3.74], compared with both the Other condition, *M* = 2.86, 95% CI [2.68, 3.03]; *t* (70) = 6.55, *p* < .001, and Control condition, *M* = 2.80, 95% CI [2.65, 2.96]; *t* (70) = 7.20, *p* < .001. There was no significant difference in the span between Other and Control conditions, *t* (70) = 0.59, *p* = .775.

#### Attempt score

An additional ‘attempt score’ was calculated to capture more variance in task performance than that provided by span, with higher scores reflecting better performance (i.e. fewer attempts required to achieve each span). For each set of sentences correctly recalled, the number of sentences in the set was multiplied by 3 if the set was passed at the first attempt, multiplied by 2 if passed on the second attempt, and multiplied by 1 if passed on the third attempt. (If not passed after three attempts, that set was awarded zero and the child did not progress.) For example, if the participant remembered a set of six sentences on the first attempt, that set would be awarded an attempt score of 18 (6 × 3). The calculation was completed for each set length and then totalled.

**FIGURE 2:**
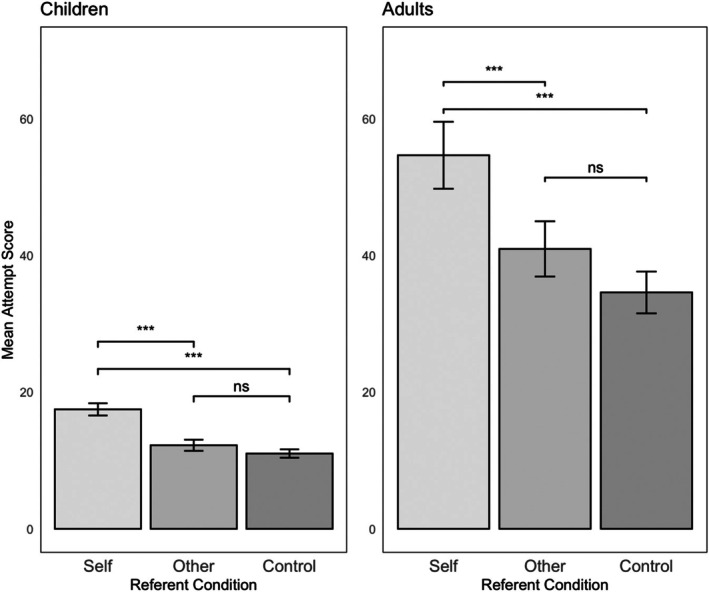
Mean attempt score for Self, Other, and Control in Experiments 1 (children) and 2 (adults). ***p < .001.

Figure 2 shows the mean attempt score across Self (Range = 35, Max = 41), Other (Range = 35, Max = 39), and Control (Range = 22, Max = 24) conditions. A repeated measures ANOVA revealed a significant main effect of Referent, *F* (2, 140) = 38.36, *p* < .001, *η*_*p*_^2^ = .35. Following the VeWM span pattern, pairwise comparisons with a Tukey adjustment revealed that participants had higher attempt scores in the ‘Self’ condition, *M* = 17.5, 95% CI [15.7, 19.3] than both the Other condition, *M* = 12.2, 95% CI [10.6, 13.8]; *t* (70) = 5.25, *p* < .001, and Control condition, *M* = 11.0, 95% CI [9.79, 12.2]; *t* (70) = 8.08, *p* < .001. There was no difference between attempt scores in the Other and Control conditions, *t* (70) = 1.65, *p* = .23.

#### Exploratory analysis

As the VeWM memory span task was adapted for the current study, performance was checked against participants' score on the standard complex span task (backward digit span). This revealed significant positive correlations between backward digit span and VeWM span in all experimental conditions (Self: *r* (69) = .288, *p* = .014; Other: *r* (69) = .326, *p* = .005; Control: *r* (69) = .248, *p* = .037).

Subsequent analyses explored the relationships between the extent to which children benefitted from the inclusion of self‐cues in the VeWM task, and their working memory and attentional abilities as measured by forward digit span, backward digit span, and the three subtests from the TEA‐Ch: selective attention (*Map Mission* task), attention control (*Opposite World* task) and sustained attention (*Score!* Task). Table 1 summarizes the performance of 7‐, 8‐ and 9‐year‐old participants on these tasks.

**TABLE 1:** Mean (*SD* in parentheses) scores on working memory and attention measures (forward digit span, backward digit span, selective attention, attentional control, and score), of 7‐, 8‐, and 9‐year‐olds.

Age (years)	*N* ^a^	Forward digit span score	Backward digit span score	Selective attention score	Attentional control score^b^	Sustained attention score
7	17 (7F, 10 M)	8.18 (*2.07*)	4.35 (*1.73*)	20.8 (*5.53*)	35.4 (*7.19*)	8.43 (*1.50*)
8	32 (19F, 13 M)	8.06 (*1.83*)	4.25 (*1.41*)	23.3 (*6.60*)	32.9 (*6.00*)	8.28 (*1.65*)
9	22 (10 F, 12 M)	9.00 (*1.95*)	5.05 (*2.06*)	29.1 (*6.86*)	30.0 (6.50)	7.53 (*2.37*)

^a^ A smaller sample of 14 7‐year‐olds, 25 8‐year‐olds, and 17 9‐year‐olds completed the Sustained Attention (Score!) task (see Participants section for details).

^b^ Note in this task only, a lower score indicates a better performance.

Two new variables, Self Advantage_Span and Self Advantage_Attempt Score were calculated. The Self Advantage_Span was calculated by subtracting Other VeWM span from Self VeWM span. The Self Advantage_Attempt Score was calculated by subtracting Other Attempt Score from Self Attempt Score. As Table 2 shows, whilst attentional task performance tended to increase with age, there was no significant correlation between either of the self‐advantage scores and scores on any of the attentional tasks.

**TABLE 2:** Correlation coefficients (Pearson's *r*) between self‐advantage scores, age, and performance on working memory and attention tasks.

	*N*	*M*	SD	1	2	3	4	5	6	7	8
1 Self advantage_span	71	0.69	0.89	–							
2 Self advantage_attempt	71	5.25	6.87	.71***							
3 Age (months)	71	101.6	10.08	.05	.05	–					
4 Forward Digit Span score	71	8.38	1.94	−.02	−.01	.19	–				
5 Backwards Digit Span score	71	4.52	1.72	−.00	−.08	.27*	.32**	–			
6 Selective attention	71	24.52	7.15	−.11	−.05	.41***	.15	.00	–		
7 Attention control^a^	71	32.63	6.67	−.09	−.03	−.33**	−.10	−.14	−.33**	–	
8 Sustained attention	56	8.09	1.87	.03	.20	−.13	.40**	.22	−.06	−.23	–

^a^ Lower score indicates better performance, so negative correlations with other attention measures indicate congruent patterns of performance.

* *p* < .05.

** *p* < .01.

*** *p* < .001.

### Discussion

Experiment 1 sought to establish, for the first time, whether working memory capacity can be directly affected by the presence of self‐cues, using a complex span task based on recall of sentence‐ending words. The results showed that children's VeWM span was significantly higher when processing self‐referent sentences than Other‐referent or control sentences. This pattern was also found when working memory capacity was assessed using an attempt score, which provided more sensitivity by applying a multiplier based on the number of attempts the child had at each set length. The children also completed measures of attention and working memory, but their performance on these tasks was not found to be related to the extent to which VeWM was improved in the self‐referent condition. Despite performance on the VeWM task being highly correlated with children's complex span measurement, the benefit to this span measure provided by self‐cues was not related to any other attention or WM measure. This suggests that self‐cues like children's own name can significantly enhance their VeWM capacity, independently of individual differences in underlying working memory, selective attention, attentional control, and sustained attention.

The novel findings of Experiment 1 demonstrate the potential of a straightforward manipulation to support children's VeWM, which could have impact in everyday life (e.g. improving performance in classroom tasks beyond the level expected on the basis of working memory capacity). Extending previous research showing that self‐cues can impact attention and elicit faster responses in visuo‐spatial tasks, the current findings show that including children's own names significantly increases VeWM capacity directly. Further, this effect was not related to any measure of individual difference in overall working memory or attentional abilities, suggesting that attentional processing support is not a compelling mechanism for the effects of self‐cues on capacity. Rather, a binding explanation is more plausible, whereby enhanced binding of self‐related information effectively chunks the information being stored in working memory, increasing capacity. However, an alternative explanation is that the children, who were within a relatively narrow age range (7–9 years), were not sufficiently varied in their working memory capacity for a reliable linear relationship with the advantage in the self‐referent condition to be detected. To address this issue and replicate the novel findings of Experiment 1, a second experiment was conducted with a sample of adults whose complex working memory span is likely to be higher than children's, with greater associated potential for range (e.g. Bayliss et al., 2003; Reynolds et al., 2022). Importantly, the inclusion of a second study with an adult sample also provided the opportunity for the magnitude of any self‐cue advantage to be compared between samples of children and adults.

## EXPERIMENT 2: OWN‐NAME EFFECTS ON VeWM IN ADULTHOOD

Following the design of Experiment 1, in Experiment 2, an adapted listening recall span task (Daneman & Carpenter, 1980) was used to assess VeWM span in Self (own name), Other (stranger name), and Control (no name) conditions in adult participants. As in Experiment 1, we expected to find a higher VeWM capacity in the Self condition than in the other two conditions, with no differences expected between the latter. We also included standard measures of working memory (forward and backward digit span) and attention (tests of selective attention, attention control, and sustained attention, adapted from the Tests of Everyday Attention [TEA], Robertson et al., 1996), to explore relationships between any advantage for the self‐referent over Other‐referent trials, and working memory and attentional abilities.

### Method

#### Participants

Fifty‐two participants (24 female) between the ages of 19 and 54 years (*M* = 27.8 years, SD = 5.63) took part in the experiment over two sessions. Participants were recruited using advertisements over social media and through the university's Graduate School. Testing for Experiment 2 was conducted online via Zoom due to the COVID‐19 pandemic. One participant was unable to attend Session 2 and therefore did not complete the attention tasks. The experiment was approved by Abertay University's Research Ethics Committee.

#### Materials and procedure

Participants were tested individually over Zoom, with their camera and microphone turned on. Session 1 began with participants completing the forward and Backward digit span tests from the Wechsler Adult Intelligence Scale‐third edition (WAIS‐III: Wechsler, 1997). The procedure for the Digit Span tests followed the instructions used in Experiment 1. The VeWM task based on Daneman and Carpenter's (1980) listening recall span task was then administered. This followed the same procedure as Experiment 1, except for some minor wording changes to suit the age group (e.g. ‘school’ replaced with ‘college’; ‘likes to play with friends’ replaced with ‘likes to play games online’). The number of words in the sentence was kept consistent across Experiment 1 and Experiment 2.

Due to the change of task delivery from in‐person to online, all three attentional processing tasks in Experiment 2 were completed during Session 2. Three attention subtests from the TEA (Robertson et al., 1996) were adapted for online testing. Selective attention was measured using the *Map Mission* subtest, as in Experiment 1. A map of Philadelphia was displayed on the screen which included symbols of similar size, and participants were instructed to locate a specific target symbol (a knife and fork). Participants were given remote access and asked to digitally circle as many targets as possible within 1 min. The total number of symbols out of a possible 80 was recorded as the score. Reflecting a difference between the TEA‐Ch and TEA, attention control was assessed using the *Visual Elevator* test rather than the *Opposite World* task. An electronic image of this test was shown on screen. In each trial, several doors were presented, accompanied by arrows pointing upwards or downwards. Participants were instructed to count the number of doors, using the arrows to determine whether they should count upwards or downwards. Two scores were generated from the test: an accuracy score (Attention control score i) reflecting the correct number of trials completed out of 10, and a time score (Attention control score ii) reflecting the time taken to correctly complete each trial (trial time divided by the number of times the participant was required to switch counting direction in those trials, to indicate the time spent transitioning between strategies). The *Elevator Counting* test (conceptually equivalent to the *Score!* Task in the TEA‐Ch) was employed to assess sustained attention. In this task, participants were instructed to imagine themselves inside an elevator with a malfunctioning floor number display. The only cue provided was a tone that played each time the elevator reached a different floor. Across seven trials, the number of tones (up to a maximum of 10) and the intervals between each tone varied. Participants were instructed to maintain a count of the number of tones they heard and indicate the final number, which represented the floor number reached. Participants' accuracy out of 7 (total number of trials) was recorded as their score.

### Results

VeWM Span and Attempt scores were calculated following the same procedures as Experiment 1.

#### Verbal working memory span

Figure 1 shows the mean verbal WM task spans for Self (Range = 6, Max = 9), Other (Range = 7, Max = 9), and Control (Range = 7, Max = 9) conditions. A repeated measures ANOVA revealed a main effect of Referent on VeWM span, *F* (2, 102) = 17.41, *p* <. 001, *η*_*p*_^2^ = .25. Pairwise comparisons between the three conditions of Referent were conducted with a Tukey adjustment. The comparisons revealed that participants had higher spans in the ‘Self’ condition, *M* = 5.96, 95% CI [5.37, 6.55], compared to both the Other condition, *M* = 5.15, 95% CI [4.60, 5.70]; *t* (51) = 3.59, *p* < .01, and Control condition, *M* = 4.77, 95% CI [4.31, 5.23]; *t* (51) = 6.02, *p* < .001. There was no significant difference in the span between the Other and Control conditions, *t* (51) = 1.98, *p* = .13.

#### Attempt score

Figure 2 shows the mean attempt score across Self (Range = 113, Max = 122), Other (Range = 125, Max = 125), and Control (Range = 108, Max = 114) conditions. A repeated measures ANOVA revealed a main effect of Referent on verbal WM attempt score, *F* (2,102) = 19.85, *p* < .001, *η*_*p*_^2^ = .28. Pairwise comparisons with a Tukey adjustment revealed that participants had higher spans in the ‘Self’ condition, *M* = 54.7, 95% CI [44.8, 64.5], than both the Other condition, *M* = 41.0, 95% CI [32.8, 49.1]; *t* (51) = 4.05, *p* = .001, and Control condition, *M* = 34.6, 95% CI [28.5, 40.7]; *t* (51) = 5.61, *p* < .001. Although there was a trend towards Other‐referent trials producing higher span scores than the control trials, this difference did not reach significance, *t* (51) = 2.30, *p* = .06.

#### Exploratory analysis

Like Experiment 1, the relationship between span as measured by the VeWM and backward digit span tests was significantly positive for all conditions (Self: *r* (50) = .433, *p* = .001; Other: *r* (50) = .348, *p* = .011; Control: *r* (50) = .390, *p* = .004). A Self‐Advantage_Span and Self Advantage_Attempt Score was calculated in the same way as Experiment 1. Exploratory correlations were conducted with the Self Advantage_Span, Self Advantage_Attempt Score, Forward Digit Span, Backward Digit Span, and the three subtests from the TEA: selective attention (*Map Mission* task), attention control (*Visual Elevator* task), and sustained attention (*Elevator Counting* task). Table 3 summarizes the mean raw scores, standard deviations, and correlations for the additional measures.

**TABLE 3:** Correlation coefficients (Pearson's *r*) between Self Advantage score, forward and backwards digit span, and the attentional measures. *N* = 52 for all measures.

	*M*	SD	1	2	3	4	5	6	7	8
1 Self Advantage_Span	0.81	1.62	–							
2 Self Advantage_Attempt	13.7	24.3	.83***	–						
3 Forward Digit Span score	11.33	2.29	.29*	.28*	–					
4 Backwards Digit Span score	8.27	2.99	.14	.19	.73***	–				
5 Selective attention	54.2	13.1	−.08	−.01	−.03	−.11	–			
6 Attention control i	8.88	1.68	−.02	.04	.26	.42**	−.19	–		
7 Attention Control ii^a^	3.48	0.95	−.14	−.16	.06	.13	−.02	.01	–	
8 Sustained attention	6.51	0.90	.06	.13	.31**	.30*	.04	.04	−.08	–

^a^ Lower score indicates better performance.

* *p* < .05.

** *p* < .01.

*** *p* < .001.

As Table 3 shows, only one attentional measure correlated with the effects of self; both Self Advantage_Span and Self Advantage_Attempt score were weakly but significantly positively correlated with forward digit span (*ps* = .04), suggesting the higher participants' forward digit span (i.e. WM storage), the more verbal WM advantage they showed in self trials.

#### Additional analyses: Comparison between Exp 1 and Exp 2 data

The children in Experiment 1 and adults in Experiment 2 completed almost identical versions of the VeWM task, differing only in the replacement of a small number of child‐focused words in the adult version. The data from Experiments 1 and 2 were therefore combined for exploratory analyses of age differences in the effects of self on VeWM span and attempt scores, on the basis that this would allow a comparison of the effects of self‐cues between two groups who differed reliably by working memory capacity. As expected, the adult sample (Experiment 2) had significantly higher Backwards Digit span scores, *M* = 8.27, 95% CI [7.63, 8.91], than the child sample (Experiment 1), *M* = 4.52, 95% CI [3.97, 5.07]; *t* (75.54) = 8.11, *p* < .001, supporting the assumption that they represented a group of higher working memory capacity.

A 2 (Age group: Children, Adults) ×3 (Referent: Self, Other, Control) mixed ANOVA on VeWM span confirmed that this measure was higher in Adults, *M* = 5.29, 95% CI [4.97, 5.62] than Children, *M* = 3.07, 95% CI [2.79, 3.35]; *F* (1, 121) = 104.3, *p* < .001, *η*_*p*_^2^ = .46, following the backward digit span pattern. There was also a main effect of Referent *F* (2, 242) = 45.67, *p* < .001, *η*_*p*_^2^ = .27, with pairwise comparisons with a Tukey adjustment confirming higher spans in the Self condition, *M* = 4.76, 95% CI [4.48, 5.03] than both the Other condition, *M* = 4.01, 95% CI [3.75, 4.26]; *t* (121) = 6.56, *p* < .001, and Control condition, *M* = 3.79, 95% CI [3.57, 4.00]; *t* (121) = 9.31, *p* < .001. The difference between the Other and Control conditions was not significant, *t* (121) = 2.20, *p* = .08. There was no interaction between Age group and Referent *F* (2, 242) = 2.36, *p* = .10, *η*_*p*_^2^ = .19 in VeWM span.

An identical mixed ANOVA with Attempt Scores as the dependent variable also revealed a significant main effect of Age group *F* (1, 121) = 88.20, *p* < .001, *η*_*p*_^2^ = .42 with a higher span for Adults, *M* = 43.4, 95% CI [38.6, 48.2] than Children, *M* = 13.6, 95% CI [9.48, 17.7]. There was a main effect of Referent *F* (2, 242) = 43.54, *p* < .001, *η*_*p*_^2^ = .27, with pairwise comparisons with a Tukey adjustment confirming higher spans in the Self condition, *M* = 36.1, 95% CI [31.8, 40.4], than both the Other condition, *M* = 26.6, 95% CI [23.0, 30.1]; *t* (121) = 6.24, *p* < .001, and Control condition, *M* = 22.8, 95% CI [20.1, 25.5], *t* (121) = 8.30, *p* < .001. There was also a significant difference between the Other and Control conditions, *t* (121) = 3.02, *p* = .009.

Using this more sensitive measure of span, the ANOVA revealed a significant interaction between Age group and Referent *F* (2, 242) = 11.00, *p* < .001, *η*_*p*_^2^ = .08. However, this was not driven by any difference in the effect of Self between age groups; simple main effects for Referent in each Age group revealed that whilst both Adults and Children show significant differences between Self and Other conditions, and between Self and Control conditions, only the Adults had a significant difference between Other and Control conditions, *t* (121) = 3.34, *p* = .003. (Note this difference reached significance in the combined Exp 1/Exp 2 analysis but not the isolated Exp 2 analysis due to the higher degrees of freedom in the former.) The children, whose Attempt scores were more compressed, showed no difference between Other and Control conditions, *t* (121) = 0.74, *p* = .74.

**FIGURE 3:**
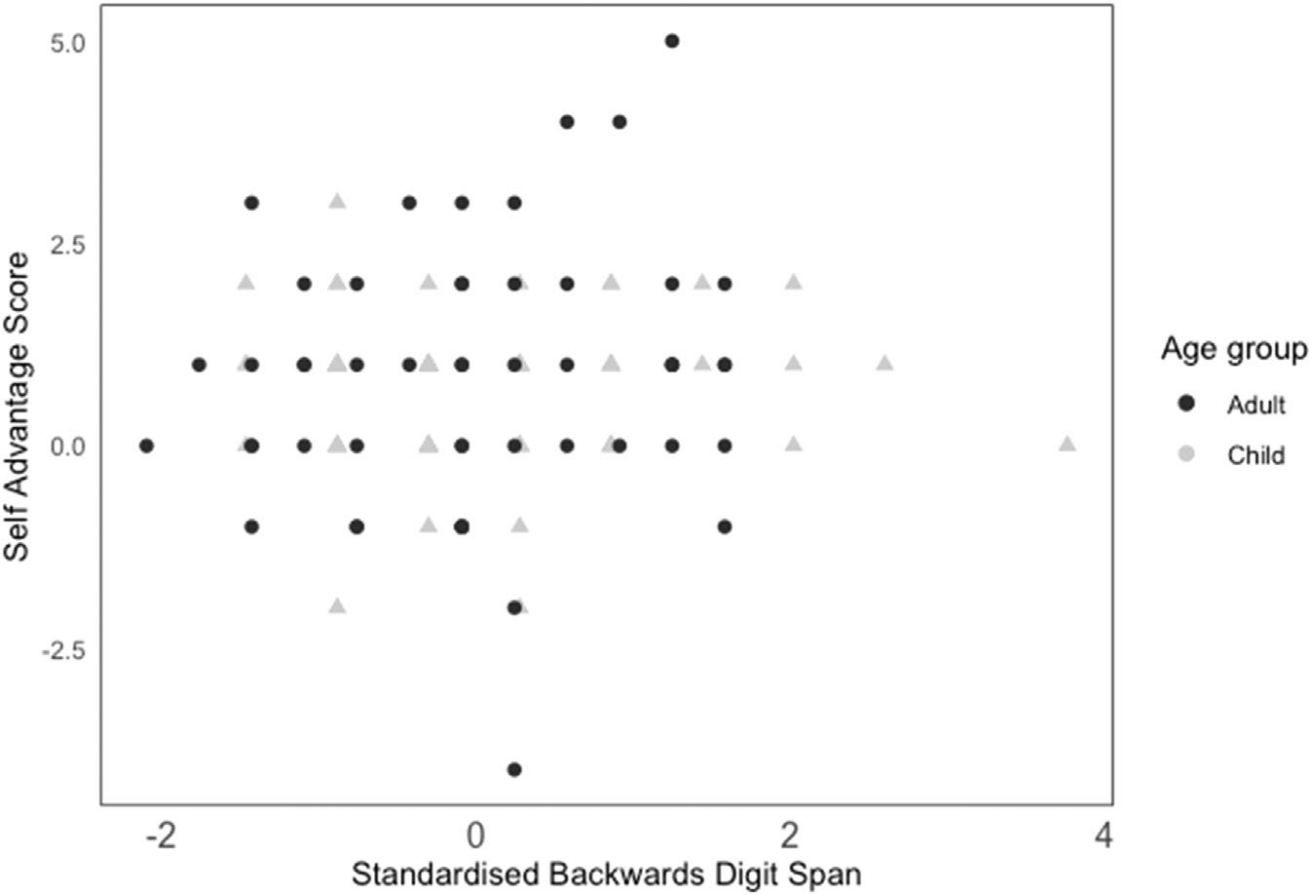
Scatterplot of self‐advantage score and backwards digit span (standardized) by group.

A final calculation examined the magnitude of the VeWM self‐advantage for participants across the child and adult samples relative to WM capacity (as measured by backward digit span). Backward digit span scores were standardized to provide a comparable measure across the two Age groups. As can be seen in Figure 3, there was no linear relationship between this measure of WM capacity and the magnitude of the self‐advantage (i.e. Self VeWM score minus Other VeWM attempt score), *r* (*121*) = .07 *p* = .41.

### Discussion

Experiment 2 explored the influence of self‐cues on adults' VeWM. The findings exactly replicated the results of Experiment 1, demonstrating that for adults as well as children, complex VeWM span can be increased by cues of self‐reference. When sentences included participants' own names, the associated span measure was higher than when the sentences included another referent name or no names. As in Exp 1, the advantage in working memory capacity elicited by self‐cues was not correlated with working memory capacity or any other measure of attentional functioning, with the exception of a weak relationship with forward digit span.

The combined Exp 1/Exp 2 analysis suggested that the effects of self‐referencing were consistent across children and adults, creating a comparable effect in the two age groups relative to both Other and Control conditions (although adults showed a greater Other‐Control difference). The effects of self on task performance were notably similar across the two experiments, despite the disparate age ranges and overall WM capacity. This provides further weight to the proposal that the mechanism underpinning the effects of self‐reference is not related to underlying attentional processing capacity, with a more plausible explanation being that it is a consequence of enhanced binding of self‐related information in working memory.

It should be noted that an isolated exception to the lack of correspondence between attentional measures and the effect of self‐cues was a weak correlation with forward digit span, which marginally exceeded the threshold of significance. Given that the forward span taps into storage (relying on the phonological loop) rather than the processing aspects of VeWM, this relationship is consistent with the proposal that self‐cues may influence storage aspects of WM by facilitating binding of information such that more can be held active. However, the relationship between forward digit span and the self‐advantage in working memory span was weak in Exp 2, and not found at all in the Exp 1 sample, so further research is needed to determine whether this pattern is spurious.

## GENERAL DISCUSSION

The present study reveals, for the first time, that self‐cues have a strong and direct effect on working memory, increasing capacity for short‐term retention. This confirms speculation based on a range of findings in earlier work on visuo‐spatial WM and mathematical tasks (Cunningham et al., 2024; D'Ailly et al., 1997; Yin et al., 2019). Strikingly, the benefit of including self‐cues was consistent across markedly different levels of working memory ability, between children and adults across the two experiments, and between individuals with differing attentional performance within each age group.

The key finding is the large and consistent effect of self‐cues on VeWM span: simply verbalizing participants' own name within the sentence enhanced their ability to successfully recall sentence contents. In terms of the underlying capacity‐increasing mechanism, previous research shows that the presentation of own‐name cues increases alertness and automatically orients attention (see Humphreys & Siu, 2016; Oberauer, 2019). But that interpretation does not fit the pattern of consistent benefits from self‐cues we observed across considerable individual variability in attentional performance, within and across both age groups. A far more compelling interpretation comes from prior work showing that self‐cues are associated with increased binding between active items (e.g. in perception, in short‐term visuo‐spatial storage, in long‐term memory episodic representations; for review, see Sui & Humphreys, 2015). This may increase the ‘chunking’ of active information as integrated representations in the episodic buffer (Hitch et al., 2020). In the span task, this would be a beneficial mechanism because the key measure is the participants' ability to keep the last word in a series of sentences active. Integrating these words into a combined unit would improve working memory capacity, but this is challenging because the words are semantically unrelated to one another. If self‐referential sentences benefit from the enhanced binding associated with self‐cues, this would facilitate the integration of the words into associated chunks, expanding participants' working memory span. Importantly, this mechanism would be expected to operate with consistency across individuals who vary by attentional processing ability and working memory capacity. Similarly consistent benefits from manipulations that target chunking in working memory, such as structured item sequences, have also been reported for people of varying underlying working memory capacity (e.g. dementia patients and healthy controls, Huntley et al., 2011). On the basis of developmental research (children vs. adults, younger vs. older adults) Gilchrest and colleagues argue that this is because whilst underlying capacity might affect the quantity of chunks that can be stored in working memory, it does not affect the quantity of information that can be chunked given the right encoding conditions (Gilchrist et al., 2008; Gilchrist et al., 2009). Enhanced binding via self‐association, acting to promote chunking in working memory, therefore, provides a plausible mechanism for the capacity‐enhancing effect of self‐cues we observed in children and adults of varying abilities.

An interesting question is the extent to which the facilitative effects identified here are specific to familiar self‐cues. As Golubickis and Macrae (2023) point out, research based on familiar cues such as one's own name may not be self‐specific. In terms of working memory, similar effects to the current findings have been reported with respect to other highly familiar stimuli, with famous faces giving rise to higher estimates of visual working memory capacity than unfamiliar faces (Jackson & Raymond, 2008). Operationalizing this, it has been proposed that the working memory system can use active elements of long‐term memory for storage, with this ‘outsourcing’ releasing capacity by reducing the storage requirements in working memory (for review of potential mechanisms see Ngiam, 2024). Accordingly, participants perform better on WM tasks when the contents include learned stimulus pairings (Bartsch & Shepherdson, 2022; Ngiam et al., 2019). Similarly, chunking and visuo‐spatial WM strategies can make use of activated long‐term memory information such as categorical rules and positional cues (e.g. clock number positions; Bartsch & Shepherdson, 2022) to reduce demands on working memory capacity.

Relating this to the current VeWM task, although familiar elements of the self‐concept could be evoked during self‐referent encoding trials, the items being held active (i.e. the words at the end of each span sentence) were not related to existing self‐knowledge at all, and indeed some sentences were deliberately nonsensical (and therefore unfamiliar) concepts. However, research on the self‐reference effect in long‐term memory shows that arbitrary information encoded with self‐cues is subject to enhanced long‐term memory storage as a result of associating it with existing self‐knowledge (see Symons & Johnston, 1997; Turk et al., 2008). This offers a mechanism through which self‐referenced items in the current task could be more easily stored as active long‐term memory representations, potentially reducing the WM storage requirements in these trials and releasing capacity. Furthermore, imagining oneself performing actions can add an enactment layer to stored representations, increasing their memorability (Allen & Waterman, 2015; Engelkamp & Zimmer, 1989). Whilst not all items in the current trial set were actionable (e.g. ‘x likes strawberries and cream’, ‘x was tired at the end of the day’), many did follow an action‐verb structure (e.g. ‘x lit the candle using a match’) so could benefit from imagined self‐enactment. Recent findings show that this type of imagined enactment can enhance working memory for verbal instructions (Yang et al., 2021, 2024), suggesting enactment could impact VeWM storage.

Building on our current findings, many opportunities open up to explore how active LTM representations could contribute to working memory, such as manipulating enactment potential within the self‐ and other‐references items, using novel self‐cues that have no pre‐existing self‐association (e.g. temporarily associated shapes instead of own names; Sui et al., 2012), and by examining how effects are modulated when cues represent familiar but non‐self‐identities (e.g. mother, best friend). Such work has the potential to reveal new theoretical insights into both the effects of self‐cues on working memory and the links between working memory and LTM. It would also be valuable for future research to explore how self‐cues impact performance across a broader range of VeWM tasks. Specifically, whilst self‐cues appear to enhance performance in the listening recall span task, where the cue is embedded in a verbal context, its effect may differ in tasks like digit span, where the self‐cue is unrelated to the sequence of numbers. Understanding the role of task relevance in modulating the effect of self‐cues would be an important direction for future work to better understand the effect of self on VeWM, and the practical applications.

As well as being of theoretical interest, the findings of the current study have potential practical benefits. They add to a small but growing body of evidence that including self‐cues like one's own name or the pronoun ‘you’ in everyday tasks with a high working memory load can measurably improve performance. This most obvious application of this finding is education, where complex processing tasks could be facilitated by self‐cues, removing potential barriers to learning (see Gathercole & Alloway, 2008). Previous research has suggested that children's performance on classroom tasks with a high working memory load can be facilitated when self‐cues are included in the materials. For example, children solve numerical word problems more quickly and accurately when these include a self‐pronoun (e.g. ‘You have 2 pencils. You have 5 pencils less than Cara. How many pencils does Cara have?’—see Cunningham et al., 2024; D'Ailly et al., 1995, 1997). Similarly, Moreno and Mayer (2000) showed that presenting task instructions with a self‐pronoun (e.g. ‘You are about to start a journey where you will be visiting different planets. For each planet, you will need to design a plant…’) improves learner's problem‐solving and information retention relative to a version with no self‐cues. These examples suggest that including self‐cues in classroom tasks can enhance performance. Whilst the current task may not be easy to translate directly into an educational activity, the findings offer an explanation for the enhancement reported by these previous studies by showing that self‐cues increase working memory capacity. When learning new tasks, working memory load often curtails performance and so the current findings help to explain why self‐cues can be applied to reduce this problem.

In conclusion, the current study is the first to show a direct effect of self‐cues on VeWM span, demonstrating that VeWM capacity can be increased by the inclusion of own‐name cues in stimuli. This may be driven by enhanced binding of self‐associated stimuli in working memory, enhancing storage capacity by reducing the quantity of active chunks, although more research is needed to investigate both the underpinning mechanisms and the extent to which they can be elicited by different self‐associated stimuli. Nonetheless, the current research demonstrates large effects of self‐cues on VeWM span in both children and adults, speaking to the robustness and consistency of this simple manipulation.

## AUTHOR CONTRIBUTIONS

**Zahra Ahmed:** Conceptualization; data curation; formal analysis; investigation; methodology; project administration; software; writing – original draft. **Janet F. McLean:** Conceptualization; data curation; formal analysis; funding acquisition; methodology; supervision; visualization; writing – review and editing. **Kevin Allan:** Methodology; writing – review and editing; conceptualization. **Sheila J. Cunningham:** Conceptualization; Funding acquisition; methodology; project administration; supervision; writing – original draft.

## CONFLICT OF INTEREST STATEMENT

The authors declare that there is no conflict of interest.

## Data Availability

The data that support the findings of this study are openly available in Open Science Framework at https://osf.io/srzfq/.

## Footnotes

^1^
Our pre‐registered analysis plan included a categorical age group variable (7, 8 or 9 years) in the ANOVA, but we were not able to run this analysis due to unequal age group sizes. For completeness, we ran an ANCOVA on both span and attempt score data, with Referent as a repeated measures factor and age in months as a continuous variable. In each measure, there was an effect of age (span: *F*(1, 209) = 7.45, *p* = .007; attempt score: *F*(1, 209) = 12.77, *p* < .001) as would be expected, but the effect of Referent remained significant after controlling for age (span: *F*(2, 209) = 23.01, *p* < .001; attempt score: *F*(2, 209) = 20.33, *p* < .001).
